# Polyp detection in the cecum and ascending colon by dye based chromoendoscopy - Is its routine use justified?

**DOI:** 10.1590/0100-6991e-20233562-en

**Published:** 2023-09-22

**Authors:** RODRIGO ALMEIDA PAIVA, FABIO LOPES QUEIROZ, PAULO ROCHA FRANÇA, BRENO XAIA MARTINS DA COSTA, LUCAS ALVES BESSA CARDOSO, DANIEL MAURICIO LONDOÑO ESTRADA, FELIPE FERREIRA DA MOTA, ANTÔNIO LACERDA

**Affiliations:** 1 - Hospital Felicio Rocho, Coloproctologia - Belo Horizonte - MG - Brasil

**Keywords:** Polyps, Colon, Endoscopy, Neoplasias Intestinais, Pólipos, Colonoscopia

## Abstract

**Introduction:**

colonoscopy is the best method for detecting polyps, with a reduction in colorectal cancer mortality of 29% and reaching 47% for distal tumors. However, it fails to demonstrate a significant reduction in proximal colon cancer mortality, and is the most common segment with interval neoplasm. The present study aimed to evaluate the impact on detection of polyps of a second sequential evaluation of cecum and ascending colon, with or without the use of indigo carmine chromoendoscopy.

**Methods:**

prospective, non-randomized clinical trial. Patients were divided into two groups. The first (G1) underwent a routine colonoscopy, followed by a second endoscopy assessment of ascending colon and cecum. The second group (G2) underwent a routine colonoscopy, followed by a second assessment of the ascending colon and cecum with indigo carmine chromoendoscopy.

**Results:**

In total, 203 patients were analyzed, 101 in the G1 and 102 in the G2. Newer polyps were identified in both groups after the second assessment with a significantly higher number of polyps detected in the patients in the G2 (p=0.0001). The number of patients who had at least one polyp in the two endoscopic assessments was significantly higher in the G2 (53 or 52% vs 27 or 26.7%, p=0.0002). In the second endoscopic assessment, the number of polyps found was also significantly higher in the G2 (50 or 76.9%) compared to the G1 (15 or 23.1%), p<0.0001.

**Conclusions:**

the second assessment with dye-based chromoendoscopy increases the detection of polyps in the ascending colon and cecum.

## RESUMO

**Introdução:**

a colonoscopia é o melhor método para detecção de pólipos, com redução da mortalidade por câncer colorretal de 29% e chegando até 47% para tumores distais. No entanto, existe falha em demonstrar redução significativa na mortalidade no cólon proximal sendo o segmento mais comum de neoplasia de intervalo. O presente estudo avaliou o impacto na detecção de pólipos em uma segunda avaliação sequencial do ceco e cólon ascendente, com ou sem o uso de cromoendoscopia com Indigo carmim.

**Métodos:**

estudo prospectivo, não randomizado. Os pacientes foram divididos em dois grupos. O primeiro (G1) foi submetido à colonoscopia de rotina, seguida de segunda avaliação endoscópica do cólon ascendente e ceco. O segundo grupo (G2) foi submetido à colonoscopia de rotina, seguida de segunda avaliação do cólon ascendente e ceco com cromoendoscopia com índigo carmim.

**Resultados:**

no total, foram analisados 203 pacientes, sendo 101 do G1 e 102 do G2. Novos pólipos foram identificados em ambos os grupos após a segunda avaliação com número significativamente maior de pólipos detectados nos pacientes do G2 (p=0,0001). O número de pacientes que apresentaram pelo menos um pólipo nas duas avaliações endoscópicas foi significativamente maior no G2 (53 ou 52% vs 27 ou 26,7%, p=0,0002). Na segunda avaliação endoscópica, o número de pólipos encontrados também foi significativamente maior no G2 (50 ou 76,9%) em relação ao G1 (15 ou 23,1%), p<0,000.

**Conclusão:**

a segunda avaliação com cromoendoscopia com índigo carmim aumenta a detecção de pólipos no cólon ascendente e no ceco.

## INTRODUCTION

Colorectal cancer (CRC) is the second neoplasm responsible for the most cancer-related deaths in the world, with a higher incidence in Europe, North America, and Oceania^1^ ^,^ ^2^, but with increasing numbers in developing countries^3^. The cumulative chance of developing colon cancer up to the age of 75 is 1.51% in men and 1.12% in women, and for rectal cancer, 1.2% in men and 0.65% in women^4^.

Prevention and early diagnosis of CRC have led to a decrease in its incidence and mortality, secondary to the detection and subsequent removal of precursor lesions during colonoscopy^5^ ^-^ ^8^. The literature indicates that up to 95% of CRC cases caused by these precursor lesions can be identified by screening methods^5^.

Screening with colonoscopy is considered the best method for detecting polyps, with a reduction in mortality from CRC of 29% and reaching up to 47% for distal tumors^5^ ^,^ ^6^. However, the use of colonoscopy fails to demonstrate a significant reduction in mortality from proximal colon cancer, this being the segment where interval neoplasms are most common^9^. This can be explained by several factors: higher prevalence of flat lesions in the ascending colon, which go more frequently unnoticed in conventional exams and with less experienced examiners; inadequate bowel preparation; accentuation of haustrations in the proximal colon, which can decrease the quality of the exam; technical limitations of the colon and colonoscopy, such as low image resolution and limited field of view^10^.

Currently, several techniques have been proposed to improve the identification of polyps in the proximal colon, including dye chromoscopy, digital chromoscopy, cap-assisted colonoscopy, routine rear view of the cecum and ascending colon, and second sequential assessment of the ascending colon.

The present study aims to evaluate the impact on polyp detection in a second sequential evaluation of the cecum and ascending colon, with or without the use of indigo carmine chromoscopy, and to discriminate which type of polyps are found by these methods.

## METHODS

We carried out this prospective, non-randomized study in a tertiary hospital in Belo Horizonte, Brazil. Patients were divided into two groups: the first (G1) underwent routine colonoscopy, followed by a second endoscopic evaluation of the ascending colon and cecum; and the second group (G2) underwent routine colonoscopy, followed by a second evaluation of the ascending colon and cecum with chromoscopy using indigo carmine dye. In both groups we considered the hepatic flexure as the distal limit. Patients were divided between groups by simple alternation. All examinations were performed by a single examiner, with high-definition endoscopes (Olympus - Evis Exera), without image magnification, and without digital chromoscopy.

Inclusion criteria were patients over 18 years of age who underwent diagnostic or screening colonoscopy and who agreed to participate in the study after signing an informed consent form. Exclusion criteria were patients with a previous history of right colectomy, incomplete colonoscopy due to technical difficulties or obstructive lesions of the distal colon, inadequate preparation of the proximal colon, active bleeding at the time of examination, previous diagnosis of inflammatory bowel disease, polyps detected by other methods (virtual colonoscopy or barium enema), colonic melanosis, incomplete colonoscopy due to hemodynamic, or anesthesiological complications.

The Research Ethics Committee of the Felício Rocho Hospital approved this study and the informed consent form.

### Technique description

Colon preparation was started the day before the exam, with a restricted liquid diet and two bisacodyl tablets (5mg) taken at 5 pm. On the day of the exam, 20% mannitol (500ml) diluted in clear juice (500ml) was used, ingested within 1 hour, approximately 5 hours before the exam. Colon preparation was evaluated according to the Aronchick scale as excellent, good, fair, poor, or inadequate^11^.

All examinations were monitored by an anesthesiologist, who performed sedation with propofol, fentanyl citrate, and/or midazolam.

At the end of the first endoscopic evaluation of the cecum and ascending colon, the patients were selected consecutively and with simple alternation to undergo a second evaluation with or without indigo carmine dye.

Indigo carmine (0.4%) was instilled directly into the working channel of the colonoscope, with a volume ranging from 20ml to 30ml, in a 60ml syringe, with the device positioned in the middle portion of the ascending colon. The syringe was always filled with air, to promote full use of the desired volume. After dye application, the air in the ascending colon was completely aspirated, allowing the dye to naturally distribute throughout the right colon, leading to adequate staining of all segments.

After identifying any polyp in the studied segment, it was immediately resected in both groups. Polyps up to 4mm were resected with biopsy forceps, those between 5mm and 10mm with a cold loop, and those larger than 10mm with a diathermic loop. Lesions larger than 20mm were resected using the mucosectomy technique.

Data were collected immediately after the examinations and included age, sex, main indication for the examination, colonic level reached, number and size of polyps found in the first evaluation, number and size of polyps found in the second evaluation, and other endoscopic findings (diverticulum, vascular ectasias, or neoplasms).

### Statistical analysis

Qualitative variables were described as absolute frequencies and percentages, quantitative variables as mean and standard deviation or median and interquartile range (Q1:Q3), depending on the type of distribution. In comparisons between groups, we used the Pearson’s chi-square asymptotic and exact tests for quantitative variables, and the Z test for proportion for qualitative variables. For quantitative variables, we used the Mann Whitney test. In the correlation analysis between the variables and the presence of polyps in the group with chromoscopy, we used the univariate and multivariate logistic regression model. The adequacy of the logistic model was evaluated using the Hosmer & Lemeshow test. The significance level used was 0.05. The software used was the SPSS version 20.0 (IBM Corp., Armonk, NY, USA) and Stata 9.1 (StataCorp., College Station, Texas, USA).

## RESULTS

From the initial sample of 219 patients, we excluded 16, six due to inadequate preparation of the ascending colon, five due to technical difficulty in reaching the cecum, two due to advanced neoplasm in the rectum that prevented the progression of the apparatus, one due to a resected specimen not recovered for histopathological study in the first evaluation, one patient with colonic melanosis and one patient with massive bleeding after polypectomy in the first evaluation. In total, we analyzed 203 patients, 101 in G1 and 102 in G2 (Flowchart 1).

**Flowchart 1:**
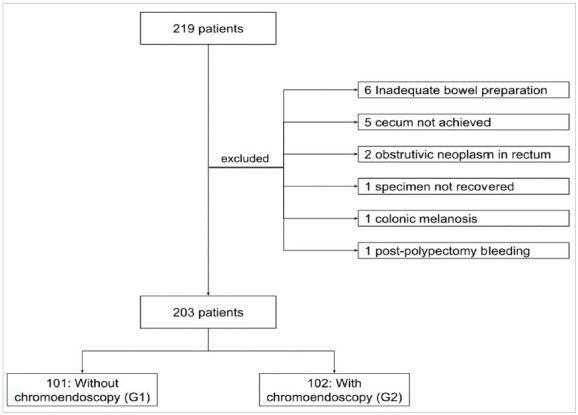
Sampling and patients.

The mean age of the patients was 59.3±12.2 years, with a predominance of females (61.6%) over males (38.4%). CRC screening was the most common indication for colonoscopy (43.8%). The second most common indication was post-polypectomy control (Table 1). There was no statistically significant difference between the groups regarding age, sex, or indication for the test (Table 2).

**Table 1:** Descriptive variables.

Variables	n=203
Age (years)	
Average ± SD	59.3 ± 12.2
Median (Q1:Q3)	59.0 (52.0:68.0)
Sex, n (%)	
Feminine	125 (61.6)
Masculine	78 (38.3)
Indication n (%)	
Hematochezia	11 (5.4)
Abdominal pain	20 (9.9)
Cold	3 (1.5)
Chronic diarrhea	5 (2.5)
Post-polypectomy follow-up	33 (16.3)
CCR screening	89 (43.8)
First-degree family history of CRC	11 (5.4)
Previous left colectomy	31 (15.3)

**Table 2:** Analysis of groups by sex and indication for the test.

Variables	G1 - No Chromoscopy	G2 - Chromoscopy	p-value
Median Age (Q1:Q3)	59.0 (52.0:67.0)	60.5 (53.0:70.3)	0.368^1^
Sex, n (%)			0.134^2^
Feminine	57 (45.6)	68 (54.4)	
Masculine	44 (56.4)	34 (43.6)	
Indication of the procedure n (%)			0.640^3^
Hematochezia	6 (54.5)	5 (45.5)	
Abdominal pain	8 (40.0)	12 (60.0)	
Constipation	1 (33.3)	2 (66.7)	
Chronic diarrhea	2 (40.0)	3 (60.0)	
Post-polypectomy control	15 (45.5)	18 (54.5)	
CACR tracking	49 (55.1)	40 (44.9)	
First degree family history	3 (27.3)	8 (72.7)	
Previous left colectomy	17 (54.8)	14 (45.2)	

^1^ **Mann Whitney test;** ^2^ **Asymptotic chi-square test;** ^3^ **Exact Pearson’s chi-square test.**

**Figure 1:**
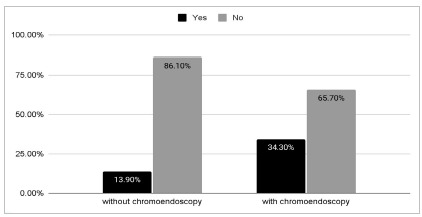
Exams with newer polyps identified in second assessment: without chromoendoscopy vs. with chromoendoscopy.

In both groups, new polyps were identified after the second evaluation (Figure 1), with a significantly higher number of polyps detected in patients who underwent the examination with chromoscopy (G2) (p=0.0001) (Table 3). Most polyps were found in the second evaluation in both groups. These polyps were smaller than 5mm (G1 66% vs G2 60%). In G1, we found five new polyps larger than 5mm, while in G2 there were 13 new polyps larger than 5mm.

**Table 3:** Characteristics of the polyp in the first vs. second review.

Variables	No chromoscopy (G1)	Chromoscopy (G2)	p-value
Identification of Polyps, 1^st^ evaluation			0.052^2^
Yes	18 (17.8)	30 (2934)	
No	83 (82.2)	72 (70.6)	
Number of polyps, 1^st^ evaluation			0.145^3^
1	12 (66.7)	26 (86.7)	
>1	6 (33.3)	4 (13.3)	
Identification of Polyps, 2^nd^ evaluation			0.001^2^
Yes	14 (13.9)	35 (34.3)	
No	87 (86.1)	67 (65.7)	
Number of polyps, 2^nd^ evaluation			0.143^3^
1	13 (92.9)	25 (71.4)	
>1	1 (7.1)	10 (28.6)	
Size of the first polyp (mm), 2^nd^ evaluation, Median (Q1:Q3)	5.0 (4.0: 6.5)	4.0 (4.0: 6.0)	0.650^1^
Size of the first polyp, 1st evaluation			0.823^2^
≤5	9 (50.0)	14 (46.7)	
>5	9 (50.0)	16 (53.3)	
Size of the second polyp, 1^st^ evaluation			1,000^3^
≤5	4 (66.7)	2 (50.0)	
>5	2 (33.3)	2 (50.0)	
Size of the third polyp, 1^st^ evaluation			0.333^3^
≤5	2 (100.0)	0 (0.0)	
>5	0 (0.0)	1 (100.0)	
Size of the first polyp, 2^nd^ evaluation			0.925^2^
≤5	9 (64.3)	22 (62.9)	
>5	5 (35.7)	13 (37.1)	
Size of the second polyp, 2^nd^ evaluation			1,000^3^
≤5	1 (100.0)	6 (60.0)	
>5	0 (0.0)	4 (40.0)	
Size of the third polyp, 2^nd^ evaluation			*
≤5	0 (0.0)	2 (40.0)	
>5	0 (0.0)	3 (60.0)	

^1^ **Mann Whitney test,** ^2^ **Asymptotic Pearson chi-square test,** ^3^ Exact Pearson chi-square test, *not enough data

The number of patients who had at least one polyp in the two endoscopic evaluations was significantly higher in the group that underwent chromoscopy (53 or 52% vs 27 or 26.7%, p=0.0002), as shown in Table 3.

In the second endoscopic evaluation, the number of polyps found was also significantly higher in G2 (50 or 76.9%) compared to G1 (15 or 23.1%), p<0.0001. There was no significant difference in the number of polyps found in the first endoscopic evaluation when using chromoscopy (35 or 57.4%) in relation to the conventional group (26 or 42.6%), p=0.097. The number of polyps added in the first and second evaluations was significantly higher in the group with chromoscopy (85 polyps or 67.5%) compared with the conventional group (41 or 32.5%), p=0.0002 (Table 4).

**Table 4:** Number of patients with polyps identified in the first and second evaluations.

Variables	No chromoscopy (G1) n=101	Chromoscopy (G2) n=102	p-value
Patients with at least 1 polyp identified during the 1^st^ evaluation (*)	13 (12.9)	18 (17.6)	0.352^1^
Patients with at least 1 additional polyp during the 2^nd^ evaluation (**)	9(8.9)	23 (22.5)	0.008^1^
Patients with at least 1 polyp in the two evaluations (intersection)	5(4.9)	12 (11.8)	0.080^1^
Patients with at least 1 polyp in the combined two evaluations (***)	27 (26.7)	53 (52.0)	0.0002^1^
Total number polyps identified during the 1^st^ evaluation (n=61)	26 (42.6)	35 (57.4)	0.097^1^
Total number polyps identified during the 2^nd^ evaluation (n=65)	15 (23.1)	50 (76.9)	<0.0001^1^
Total number of polyps identified during the 1^st^ and 2^nd^ evaluations (n=126)	41 (32.5)	85 (67.5)	0.0002^1^

^1^ Z Proportion Test; *Patients identified only in the first assessment; **Patients identified only in the 2 ^nd^ assessment; ***Patients identified only in the first evaluation + only in the second evaluation + intersection.identificados somente na primeira avaliação + somente na segunda avaliação + interseção.

Considering the polyps’ histopathology (hyperplastic and adenomas with low-grade dysplasia), we observed no difference between the groups in both the first and second evaluations (Table 5). In none of the groups there was any polyp identified with high-grade dysplasia or with invasive neoplasia.

**Table 5:** Histopathological analysis of polyps identified in the right colon and cecum.

Variables	No chromoscopy (G1) n=101	Chromoscopy (G2) n=102	p-value
HA at first assessment			0,159^1^
hyperplastic	2 (10,5)	8 (27,6)	
low-grade adenoma	17 (89,5)	19 (65,5)	
high-grade adenoma	0 (0,0)	2 (6,9)	
adenocarcinoma	0 (0,0)	0 (0,0)	
HA in the second evaluation			0,294^1^
hyperplastic	2 (14,4)	10 (21,2)	
low-grade adenoma	7 (63,6)	22 (68,8)	
high-grade adenoma	0 (0,0)	0 (0,0)	
adenocarcinoma	0 (0,0)	0 (0,0)	
HA of the transverse colon			1,000^1^
Hyperplastic	4 (36,4)	6 (40,0)	
low-grade adenoma	7 (63,6)	9 (60,0)	
high-grade adenoma	0 (0,0)	0 (0,0)	
adenocarcinoma	0 (0,0)	0 (0,0)	
HA of the descending colon			0,378^1^
hyperplastic	5 (27,8)	7 (30,40	
low-grade adenoma	11 (61,1)	16 (69,6)	
high-grade adenoma	2 (11,1)	0 (0,0)	
adenocarcinoma	0 (0,0)	0 (0,0)	
HA of the rectum			0,244^1^
hyperplastic	16 (80,0)	7 (50,0)	
low-grade adenoma	3 (15,0)	5 (35,7)	
high-grade adenoma	1 (5,0)	2 (14,3)	
adenocarcinoma	0 (0,0)	0 (0,0)	

^1^ **Exact Pearson’s chi-square test. HA: Histopathological analysis.**

We also performed an analysis of the other colonic segments, in addition to the cecum and ascending colon. Comparison of the groups with and without chromoscopy showed no statistical difference regarding the number of polyps found or their size (Table 6).

**Tabela 6:** Identificação de pólipos nos demais segmentos cólicos.

Variáveis	Sem cromoscopia(G1) n=101	Cromoscopia (G2) n=102	Valor-p
Identificação de Pólipos transverso			0,694^1^
Sim	12 (11,9)	14 (13,7)	
Não	89 (88,1)	88 (86,3)	
Quantidade de Pólipos transverso			1,000^2^
1	11 (91,7)	12 (85,7)	
2	1 (8,3)	2 (14,3)	
Tamanho de Pólipos transverso			1,000^1^
≤5	6 (50,0)	7 (50,0)	
>5	6 (50,0)	7 (50,0)	
Identificação de Pólipos descendente			0,349^1^
Sim	22 (21,8)	28 (27,5)	
Não	79 (78,2)	74 (72,5)	
Quantidade de Pólipos descendente			0,085^2^
1	20 (90,9)	19 (67,9)	
>1	2 (9,1)	9 (32,1)	
Tamanho de Pólipos descendente			0,361^1^
≤5	9 (40,9)	8 (28,6)	
>5	13 (59,1)	20 (71,4)	
Identificação de Pólipos reto			0,556^1^
Sim	19 (18,8)	16 (15,7)	
Não	82 (81,2)	86 (84,3)	
Tamanho de Pólipos reto			0,149^1^
≤5	14 (73,7)	8 (50,0)	
>5	5 (26,3)	8 (50,0)	
Quantidade de Pólipos reto			0,677^2^
1	16 (84,2)	12 (75,0)	
>1	3 (15,8)	4 (25,0)	

^*1*^ *Teste Qui-quadrado de Pearson assintótico;* ^*2*^ *Teste Qui-quadrado de Pearson exato.*

The results of the multivariate analysis showed that only the variables age and gender were associated with the variable absence and presence of polyps. For each additional year in the patient’s age, the risk of having a polyp increases by 1.05. The risk of males having a polyp is 2.66 compared with females. The logistic regression model was adequate for the data (p=0.278)

## DISCUSSION

To reduce the incidence and mortality of CRC, it is essential that colonic polyps be removed at screening colonoscopies. Even with all the technological advances, a significant portion of these injuries remain unidentified. Digital chromoscopy has been proposed as a less laborious and faster alternative when compared with dye chromoscopy, but it has not demonstrated significant gains in polyp detection for the purposes of CRC prevention^12^ ^,^ ^13^.

The use of indigo carmine has already been described as a way of optimizing colonoscopy and examining specific areas of the colon suspected of having any alteration^14^. Dye chromoscopy can also be used throughout the colon and rectum, from the beginning of the exam, called panchromoscopy^20^. This technique is associated with a slight increase in the total exam time and an increase in polyp detection^15^. Panchromoscopy can also be used in patients with inflammatory bowel disease, where it is used to improve the accuracy of pathological findings in direct biopsies.

We opted for chromoscopy with dye in a specific segment of the colon due to the easy anatomical characterization of the cecum and ascending colon (appendicular ostium, ileocecal valve, and hepatic flexure) and mainly because it is a segment where the protective value of colonoscopy is questioned. One of the explanations for this possible failure of protection is the predominance of flat lesions in the ascending colon, resulting in a higher incidence of interval cancer in the proximal colon, through the serrated pathway of carcinogenesis^16^. The fact that these lesions are generally flat increases the possibility of going unnoticed in conventional exams^17^.

In the present study, when summing the number of polyps (first and second evaluations), we found more than twice as many polyps in the chromoscopy group, a statistically significant difference. Lapalus et al.^18^ described similar findings in a study involving panchromoscopy, showing an increase in the detection of polyps only in the proximal colon. It must be considered, however, that only a second evaluation of the ascending colon and cecum without additional resources was also able to demonstrate an increase in the number of polyps diagnosed and removed, one of the possible reasons for this finding being the longer colonoscopic evaluation of these patients. However, it is important to consider that this study may have been influenced by the so-called “Hawthorne effect”, in which examiners may have shown improved performance due to involvement with new techniques being tested.

Nonetheless, in the present study, this increase was significant only in the group of patients undergoing colonoscopy with chromoscopy. Park et al.^19^ also showed an increase of 17.9% after a second evaluation, without additional resources, compared with 41.6% in the group undergoing chromoscopy. The same was demonstrated in relation to the number of patients with polyps in the two summed evaluations. After the second evaluation, in patients in whom at least one additional polyp was identified, we observed a greater number of patients with polyps in the group of patients undergoing colonoscopy with chromoscopy (23 vs nine, p=0.008). Patients who had at least one polyp in the two combined evaluations were also observed in greater numbers in the group with chromoscopy (53 vs 27, p=0.0002). A recent systematic review involving seven studies showed an increase in the total number of polyps diagnosed after dye chromoscopy, in addition to an increase in the number of patients with at least one diagnosed polyp, favoring the use of indigo carmine^20^. 

The proposal to use chromoscopy with indigo carmine is a simple and low-cost complementary method, bringing a summation effect to an examination that until then would be complete. Its potential in the detection of serrated-type flat lesions stands out, with its own pathway of carcinogenesis, involving mutation in RAS and BRAF, located mainly in the ascending colon, and very similar to the surrounding mucosa in most cases^21^.

The results of the present study show a gain in polyp detection when indigo carmine chromoscopy is used after conventional examination of the cecum and ascending colon, without additional risks, but with increased time. More prospective studies on the subject are awaited for a better understanding and to define its indication as CRC prevention.

## CONCLUSION

The second evaluation with contrast-enhanced chromoscopy showed a statistically significant increase in the detection of polyps in the ascending colon and cecum when compared with the second evaluation with conventional colonoscopy. In addition, performing a second assessment also increases polyp detection compared with not performing it.
